# Taxonomic note of *Parnassia* (Celastraceae) in China II: population surveys reveal that *P.guilinensis* is conspecific to *P.xinganensis*

**DOI:** 10.3897/phytokeys.172.62749

**Published:** 2021-02-10

**Authors:** Xiao-Song Dai, Dao-Zhang Min, Bo Yang, Ding Wu, Bo Li

**Affiliations:** 1 Jiangxi Key Laboratory of Plant Resources and Biodiversity, Jingdezhen University, Jingdezhen 333000, China Jingdezhen University Jingdezhen China; 2 School of Life Sciences, Nanchang University, Nanchang 330031, China Nanchang University Nanchang China; 3 Research Centre of Ecological Sciences, College of Agronomy, Jiangxi Agricultural University, Nanchang 330045, China Jiangxi Agricultural University Nanchang China

**Keywords:** Endemic species, morphology, staminode, synonymy, taxonomy

## Abstract

Based on investigation of populations of *Parnassiaguilinensis* and *P.xinganensis*, examination of herbarium specimens (including types), as well as consultation of protologues and distributions, *P.guilinensis* is hereby reduced to a synonym of *P.xinganensis*. *P.xinganensis* is endemic to northeastern Guangxi Province of China and characterized by having elliptic to ovate leaves and staminodes 3–5-branched with globose glands. Field photographs and an updated morphological description of *P.xinganensis* are provided.

## Introduction

*Parnassia* L. consists of small, glabrous and rosulate perennial herbs, with several morphologically distinguishable traits, including a solitary, terminal, bisexual and pentamerous flower borne on an unbranched scape; five showy staminodes; and one-by-one stamen movement (Ku 1987; Ku and Hultgård 2001; Sandvik and Totland 2003; Ren 2010; Armbruster et al. 2014; Ren and Bu 2014). Species of *Parnassia* predominantly occur in arctic and alpine regions of Europe, Asia, and North America with a center of diversification in mountainous areas in Pan-Himalaya and southwest China (Phillips 1982; Ku 1987; Ku and Hultgård 2001; Wu et al. 2003; Simmons 2004; Wu 2005). According to the most updated checklist, *Parnassia* contains 61 species, 2 subspecies, 11 varieties and 1 form (Shu et al. 2017). However, results from several taxonomic investigations after Shu et al. (2017) have changed the number of species in the genus. These changes include: P.lanceolatavar.oblongipetala T.C. Ku was reduced to a synonym of *P.yunnanensis* Franch. (Shu and Zhang 2017), *P.venusta* Z.P. Jien, *P.degeensis* T.C. Ku and *P.kangdingensis* T.C. Ku were reduced to synonyms of *P.cacuminum* Hand.-Mazz. (Shu and Zhang 2017), *P.chengkouensis* T.C. Ku and *P.dilatata* Hand.-Mazz. were reduced to synonyms of *P.wightiana* Wall. ex Wight et Arn. (Wang et al. 2018), *P.brevistyla* (Brieg.) Hand.-Mazz. and *P.leptophylla* Hand.-Mazz. were reduced to synonyms of *P.delavayi* Franch. (Yu et al. 2018), *P.tibetana* Z.P. Jien ex T.C. Ku, P.nubicolasubsp.occidentalis Schönbeck-Temesy and P.nubicolavar.nana T.C. Ku were reduced to synonyms of *P.nubicola* Wall. ex Royle (Ma et al. 2020). Meanwhile, some new species were described, including *P.zhengyuana* M.X. Ren et J. Zhang and *P.simianshanensis* M.X. Ren, J. Zhang et Z.Y. Liu (Zhang et al. 2019).

**Figure 1.**
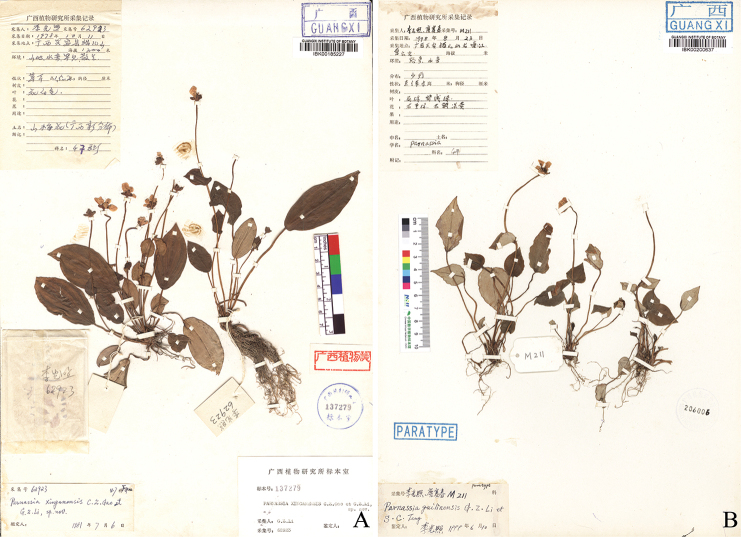
Type specimens of *Parnassiaxinganensis* (**A**) and *P.guilinensis* (**B**).

**Figure 2.**
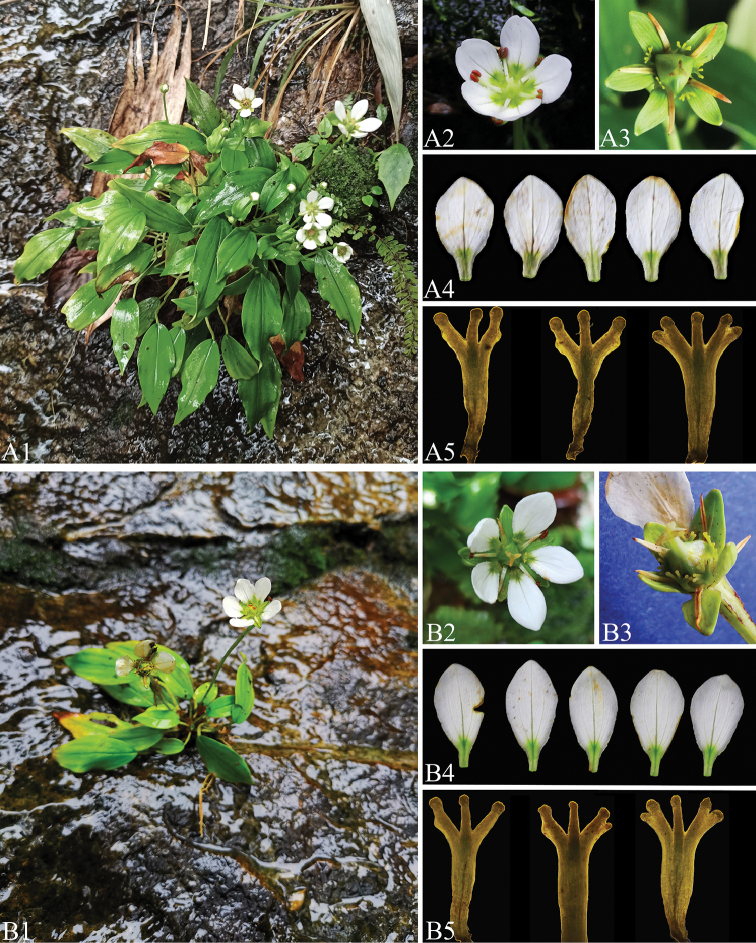
Morphological comparisons between *Parnassiaxinganensis* (**A1–A5**) and *P.guilinensis* (**B1–B5**) collected from their type localities of Mao’er Mountain at altitudes of 1200 m and 580 m, respectively **A1, B1** habitat **A2, B2** flower **A3, B3** calyces, staminodes and ovary **A4, B4** petals **A5, B5** variation of staminodes.

In the taxonomy of *Parnassia*, the shape of basal leaves, the characteristics of petals (entire or flat, divided into lobes or filiform rays, respectively), and the shape of staminodes (i.e., number and depth of staminode branches, shape of staminode lobes, with globose glands at apex or not) were considered to be of great significance in species delimitation (Ku 1987; Ku and Hultgård 2001; Wu 2005). *P.guilinensis* G.Z. Li et S.C. Tang (Tang and Li 1999) and *P.xinganensis* C.Z. Gao et G.Z. Li (Gao and Li 1983) were described based on specimens collected from the same mountain: Mao’er Mountain, Xing’an County, Guilin City, Guangxi Province, China. The type specimen of *P.xinganensis* (11 December 1978, *G.Z.Li 62923*) was collected at an altitude of 1200 m on the mountain while that of *P.guilinensis* (23 August 1998, *G.Z.Li and S.C.Tang M93*) was collected at 580 m. Tang and Li (1999) noted that *P.guilinensis* was morphologically similar to *P.xinganensis* but could be distinguished from the latter by the staminodes being 5-lobed and the apex of lobes with globose glands, whereas the staminodes of *P.xinganensis* are 3- or rarely 4-lobed without globose glands at the lobe apex. In both *Flora Reipublicae Popularis Sinicae* (Ku 1995) and *Flora of China* (Ku and Hultgård 2001), the staminodes of *P.xinganensis* were described as eglandular. However, when examining the type specimen of *P.xinganensis* (Fig. 1), we found that the apex of the staminode lobes obviously bear globose glands and the number of lobes range from three to five, strongly indicating that *P.xinganensis* and *P.guilinensis* are the same species. In order to clarify the relationship of the two sympatric taxa, we visited the Mao’er Mountain in September and October of 2020 and conducted population surveys of *Parnassia* species from low to high altitudes. Population observations clearly revealed that there are no differences among individuals at 580 m and 1200 m (Fig. 2), confirming that the later described species *P.guilinensis* is conspecific with the former one, *P.xinganensis*. We thus reduce *P.guilinensis* to a synonym of *P.xinganensis*, and provide an updated description of *P.xinganensis* based on population observations.

## Materials and methods

Type specimens of *P.guilinensis* and *P.xinganensis* deposited at IBK, as well as other *Parnassia* specimens collected from Mao’er Mountain, Xing’an County, Guangxi Province, China, preserved in GXMG, IBK, IBSC, KUN and PE (acronyms according to Thiers 2020+) were carefully examined under a stereo dissecting microscope (Stereo Zoom Leica S8 APO, Leica Microsystems 2017). Morphological traits were measured using a ruler and a micrometer based on both herbaria and fresh materials. Field investigations were carried out during September to October of 2020 in Mao’er Mountain and some individuals of *P.xinganensis* collected from different altitudes were transplanted to the greenhouse at Jingdezhen University for further observation and to be photographed. Voucher specimens were deposited at the herbarium of Jingdezhen University.

## Taxonomic treatment

### Parnassia xinganensis

Taxon classificationPlantae

C.Z. Gao & G.Z. Li, 1983: 19.

B83D381A-18FD-5BCE-A3B0-7F3173C69FD4

Figs 1 , 2

#### Type.

China. Guangxi: Guilin City, Xing’an County, Mao’er Mountain, streamsides in valleys, alt. 1200 m, 11 December 1978, *G.Z.Li 62923* (holotype: IBK00185227!; isotype: IBK00200466!).

=*Parnassiaguilinensis* G.Z. Li & S.C. Tang, syn. nov. Type: China. Guangxi: Guilin City, Xing’an County, Mao’er Mountain, streamsides, alt. 580 m, 23 August 1998, *G.Z.Li & S.C.Tang M93* (holotype: IBK00200636!).

#### Description.

Perennial herbs, glabrous. Rhizome sympodial, robust. Stems 1–8, 5–20 cm tall, usually with 1 cauline leaf near middle. Basal leaves (4–) 8–13 (–22); petiole (1–) 4.5–6.5 (–9.5) cm long; leaf blade elliptic, obovate-elliptic, oblong-ovate to ovate, abaxially gray-white, adaxially green, (1–) 3–5.5 (–7.5) × (0.8–) 1.5–2.5(–3) cm, inconspicuously 5–7-veined on both surfaces, midvein prominent, base rounded, subtruncate to cuneate, apex obtuse to acute. Cauline leaf sessile, amplexicaul, ovate or ovate-triangular, 0.9–2.4 × 0.5–1.6 cm. Flowers 1.5–2.3 cm in diam.; hypanthium shortly campanulate or inconspicuous. Sepals green, elliptic to ovate, 3.7–5.2 × 2.3–3.7 mm, 5-veined, margin entire, apex obtuse. Petals spreading, white, elliptic to broadly obovate, 8.5–12 × 6.8–8.3 mm, 5-veined basally, base attenuate into a claw, 2–3 mm long, margin entire or slightly undulate, apex rounded, obtuse or emarginate. Anthers ellipsoid; filaments 2–7 mm long; staminodes flat, 3–3.5 mm long, 3–5-branched to middle, branchedes globose glandular at apex. Ovary superior, greenish, ovoid, slightly sunken into hypanthium; styles short, ca. 1–1.5 mm long; stigma 3-lobed, lobes oblong, spreading. Capsule ovoid, trigonous, 5–10 mm long, 3-valved. Seeds minute, oblong, ca. 1 mm long.

#### Phenology.

Flowering – late June to November; fruiting – August to December.

#### Distribution and habitat.

The species is endemic to northeastern Guangxi Province (recorded only in Xing’an County and Ziyuan County), China, and grows in clefts of the moist rocks along streams or under waterfalls, at an elevation of 400–1350 m.

#### Additional specimens examined.

China. Guangxi: Guilin City, Xing’an County, Mao’er Mountain, streamsides, moist rocks in valleys, alt. 580 m, 23 August 1998, *G.Z.Li & S.C.Tang M211* (IBK00200637!); under forests, alt. 611 m, 29 September 2014, *Xing’an Expedition 450325140929020LY* (GXMG0110865!); Ziyuan County, Mao’er Mountain, on rocks under forests, near streams, 6 December 1980, *G.Z.Li 10120* (IBK!).

#### Conservation status.

At present, *P.xinganensis* has been reported only from two counties in northeastern Guangxi Province of China. Based on our field investigations, there are numerous mature individuals and young seedlings which could be easily discovered along streams and under waterfalls, indicating the population survives and regenerates well. Additionally, the Mao’er Mountain has been projected to a national nature reserve of China in 2003, and ranks among one of the earliest national nature reserves founded in Guangxi Province. It is apparent that the species will not be severely affected by human activities, thus we propose to list *P.xinganensis* as Least Concern (LC) according to the IUCN Red List categories and criteria (IUCN 2017).

## Supplementary Material

XML Treatment for Parnassia xinganensis
